# In vitro analysis of expression vectors for DNA vaccination of horses: the effect of a Kozak sequence

**DOI:** 10.1186/1751-0147-50-44

**Published:** 2008-11-04

**Authors:** Guðbjörg Ólafsdóttir, Vilhjálmur Svansson, Sigurður Ingvarsson, Eliane Marti, Sigurbjörg Torsteinsdóttir

**Affiliations:** 1 Institute for Experimental Pathology, University of Iceland, Keldur, Reykjavík, Iceland; 2 Department of Clinical Veterinary Medicine, Vetsuisse Faculty, University of Berne, Switzerland

## Abstract

One of the prerequisite for developing DNA vaccines for horses are vectors that are efficiently expressed in horse cells.

We have analysed the ectopic expression of the human serum albumin gene in primary horse cells from different tissues. The vectors used are of pcDNA and pUC origin and include the cytomegalovirus (CMV) promoter. The pUC vectors contain CMV intron A whereas the pcDNA vectors do not.

Insertion of intron A diminished the expression from the pcDNA vectors whereas insertion of a Kozak sequence upstream of the gene in two types of pUC vectors increased significantly the *in vitro *expression in primary horse cells derived from skin, lung, duodenum and kidney.

We report for the first time the significance of full consensus Kozak sequences for protein expression in horse cells *in vitro*.

## Background

DNA vaccines have attracted great interest since they induce strong and lasting humoral and cellular immune response in experimental animals. Their ability to modulate the immune response and to shift it from Th2 to Th1 holds a promise for treatment of allergies and cancer [1,2]. In large animals and humans DNA vaccines have, however, not lived up to this expectation. Their major drawback is low and short lived immune response [3,4]. One of the reasons for this is thought to be due to limited expression of the gene product involved and few activated antigen presenting cells. It is therefore important to improve the efficacy of expression in the cells of the relevant animal [5,6].

Virus-based vector vaccines have been quite effective in attaining protection against several viral diseases in horses such as influenza [7,8], West Nile fever [9-12] and equine viral arteritis [12,13]. Some of those vaccines have been licensed [7,9]. With plasmid based DNA vaccination of horses, protection has been achieved against West Nile virus with a single immunisation [14]. However, the potency of this type of genetic vaccines still needs to be improved for obtaining an adequate immune response without using extreme means of injection such as sensitive sites and too many boosts [9,15].

In vectors used for DNA vaccines strong promoters are used to give the maximum expression of antigens. The most commonly used is the cytomegalovirus immediate early gene promoter (CMV-IE) [16,17]. The strongest expression is generally obtained when the full length, enhanced CMV-IE promoter is used, including the first intron from the IE1 gene (intron A) [18-20].

A Kozak sequence adjacent to the ATG start codon greatly increases the efficiency of translation and hence overall expression of the gene product. It functions by slowing down the rate of scanning by the ribosome and improving the chance of it recognising the start of translation at the AUG start codon. For optimal expression it is recommended to use the full consensus (GCC)GCC A/G CC ATG G [21,22].

Our efforts to Th1 focus the immune response of horses by vaccinating them with vectors of pcDNA origin resulted in low immune response [23]. We therefore tried to improve the expression from the vectors with a Kozak sequence and an intron A. Insertion of the Kozak sequence increased the expression in all the cells whereas addition of the intron A decreased the expression.

## Methods

### 2.1. Construction and purification of vectors

**Figure 1:**
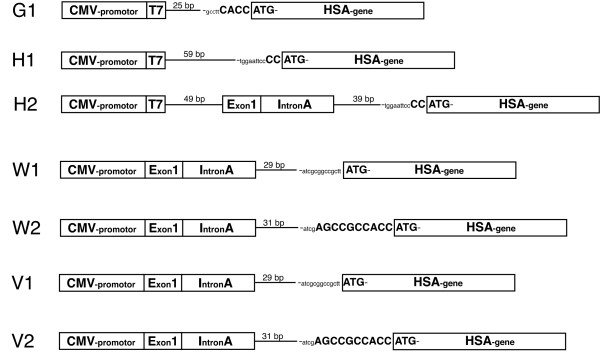
**Linearized format of the vectors used in the study**. G1: pcDNA3.1/GS-HSA, H1: pcDNA3.1/V5-His+HSA, H2: pcDNA3.1/V5-His+HSA with Intron A insert from VR1012, W1: gWIZ+HSA, W2: gWIZ+HSA with Kozak, V1: VR1012+HSA and V2: VR1012+HSA with Kozak. CMV-promoter: Human cytomegalovirus immediate early I promoter/enhancer, T7: T7 promoter priming site, 25–59 bp: Variable number of base pairs in vector backbone, Exon 1: CMV Exon 1, Intron A: CMV Intron A, HSA gene: Human serum albumin gene. The whole and semi Kozak sequences are shown with capital letters.

Origin and modification of vectors is shown in table 1 and figure 1. The HSA gene (1822 nucleotides, database no NM000477) was amplified by polymerase chain reaction (PCR) from pcDNA3.1/GS-HSA (G1) (Invitrogen), digested with EcoRI and XhoI and ligated with T4 DNA ligase into pcDNA3.1/V5-His (Invitrogen) (H1). The gene was amplified using primers 5'-GGTGTGAATTCCATGAAGTGGGTAACCTTTAT-3' and 5'-GGTGTCTCGAGCGTAAGCCTAAGGCAGCTTGA-3' and cloned in frame with V5 epitope and polyhistidine tag. The CMV intron A was amplified by PCR from VR1012 (Vical) (V), using 5'-CAGTTAAGCTTCGCAGAGCTCGTTTAGTGA-3' and 5'-CAGTTGGATCCAGTGTCGACGACGGTGAC-3', primers that included splice sites. The PCR product was digested with BamHI and HindIII (Fermentas) and ligated into H1 between the promoter and the HSA gene to make vector H2. Different from the parental vector V there are additional 111 nucleotides between the CMV promoter and intron A in vector H2 (Figure 1). The HSA gene, V5 epitope and 6His tag were amplified by PCR from H1 (pcDNA3.1/V5-His+HSA), digested with BamHI and NotI and ligated into V (VR1012) and gWIZ (W) (Gene Therapy Systems, Inc.) plasmids with or without a typical Kozak sequence. The translation initiation site of HSA was modified towards consensus Kozak sequence GCCACCATG when the gene was amplified from H1. The HSA gene, V5 and His6 tags were amplified using 5'-GGTATGCGGCCGCTTATGAAGTGGGTAACCTTTAT-3' without Kozak or using 5'-GTATGCGGCCGCCACCATGAAGTGGGTAACCTTTAT-3' with Kozak sequence and 5'-CGCTAGGATCCAATCAATGGTGATGGTGATGATG-3'. Taq DNA Polymerase (New England BioLabs) was used for PCR amplifications. The PCR products and DNA digested with restricted endonucleases were extracted and purified from agarose gel with QIAEX II kit according to suppliers protocol (QIAGEN).

Selected clones were grown in LB broth (DIFCO) containing the appropriate antibiotics. The plasmids were propagated in the DH5α strain of *E. coli*, harvested and purified by QIAGEN Plasmid Midi Kits according to the suppliers protocol (QIAGEN). Verifying the presence of the HSA gene and the intron A in the plasmids was done with restriction enzymes; amplified by PCR; and sequenced with universal and specific primers. The Kozak sequence was verified by DNA sequencing using BigDye Terminator v3.1 Cycle Sequencing Kit (Applied Biosystems). The pBudCE4.1/lacZ/CAT vector was purchased from Invitrogen.

### 2.2. Cell cultures

Primary horse cells were derived from lung and kidney tissue of a horse fetus and skin and duodenum of foals. The lung, kidney and skin cells were fibroblast like but very different in morphology and growth rate. The duodenum cells had endothelium morphology. The lung, kidney, skin and the African green monkey kidney cells (COS-7) (ATCC) were propagated in Dulbecco's MEM (DMEM) (Invitrogen, GIBCO) supplemented with 2 mM glutamine, 100 IU/ml penicillin, 100 IU/ml streptomycin and 10% fetal bovine serum (Invitrogen, GIBCO) referred to as DMEM growth medium. The duodenum was cultured in CS-S medium for endothelial cells (Sigma) supplemented with 2 mM glutamine, 100 IU/ml penicillin, 100 IU/ml streptomycin, 1% endothelial growth factor (Sigma) and 20% FCS. The primary cells were not used in higher than 10^th ^passage.

### 2.3. Transfection

**Figure 2:**
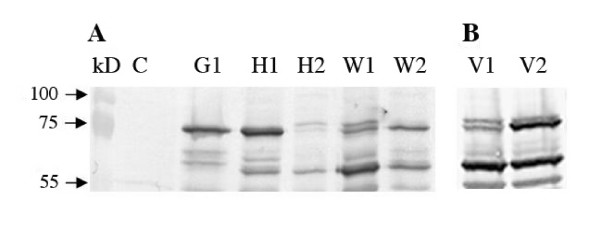
**Expression of HSA gene on different vectors in COS-7 cells**. COS-7 cells were transfected with HSA vectors using Lipofectamine 2000, cultured for 48 h, harvested and applied to Western blot. Control (C) cells treated the same way without DNA. (A) HSA vectors: pcDNA3.1/GS (G1), pcDNA3.1/V5-His (H1), pcDNA3.1/V5-His with intron A (H2), gWIZ (W1) and gWIZ with Kozak (W2). (B) HSA vectors:VR1012 (V1) and VR1012 with Kozak (V2). The vectors were tested at least three times.

**Figure 3:**
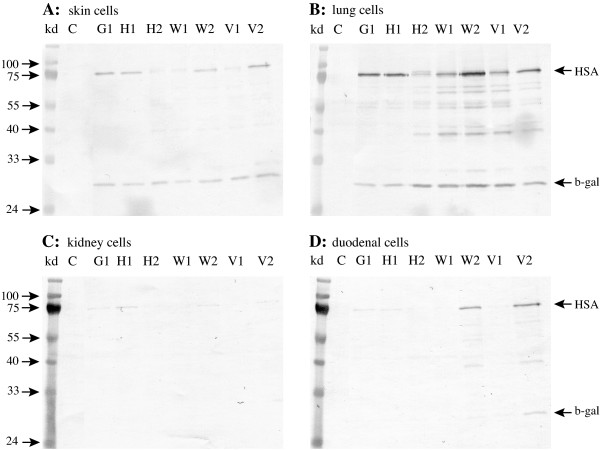
**Expression of HSA gene on different vectors in primary equine skin (a), lung (b), kidney (c) and dudenum (d) cells**. The cells were transfected simultaneously with HSA vectors and pBudCE4.1/lacZ/CAT control vector using Lipofectamine 2000, cultured for 48 h, harvested and applied to Western blot. Control (C) cells treated the same way without DNA. HSA vectors: pcDNA3.1/GS (G1), pcDNA3.1/V5-His (H1), pcDNA3.1/V5-His with intron A (H2), gWIZ (W1), gWIZ with Kozak (W2), VR1012 (V1) and VR1012 with Kozak (V2). The 75 kDa HSA band and the 30 kD CAT band from the pBudCE4.1/lacZ/CAT plasmid is indicated. The vectors were tested at least 3 times in each cell line.

The expression of HSA was tested by transfection of COS-7 cells using Lipofectamine 2000 (Invitrogen) following the protocol recommended by the manufacturer. Briefly the cells were cultured in monolayer to 90–95% confluency in DMEM growth medium in 12-well plate (NUNC). Lipofectamine 2000 was diluted 1: 25 in Opti-MEM (Invitrogen, GIBCO) (85 μl) and incubated 5 min at room temperature (RT). DNA was diluted to 1.35 μg/ml in Opti-MEM (85 μl) mixed with the Lipofectamine 2000 solution, incubated 20 min at RT and then added to the cells. Transfection was performed in culture medium without antibiotics for 48 hrs (Figure 2). Transfection for 24 hrs gave similar results (data not shown). Cells treated the same way with Lipofectamine 2000 but without DNA served as negative controls. The primary horse cells were transfected in the same way except two types of plasmids instead of one were used for transfection. The pBudCE4.1/lacZ/CAT vector (Invitrogen) was used to control the transfection. The vectors with the HSA gene, 1,35 μg/ml and pBudCE4.1/lacZ/CAT 0,6 μg/ml were mixed in 100 μl Opti-MEM. The vectors were tested at least three times in the cell lines and for obtaining the results shown in figure 3 the vectors were transfected into all the cells at the same time point.

### 2.4. Western blot

The expression of HSA and CAT was monitored in Western blot. SDS-PAGE was done in the Mini-protean II system from Bio-Rad according to manufactures instructions. In short, transfected cells and control cells were boiled (1:1 vol) in 2× reducing sample buffer and applied to a denaturing 12% separation gel followed by a transfer to Immobilon-P membrane (Millipore) using semi-dry MilliBlot Graphite Electroblotter (Millipore). Membranes were incubated overnight at 4°C with 1:5000 mouse monoclonal antibody against V5 (Invitrogen) then 1 hr at RT with goat anti-mouse IgG conjugated to alkaline phosphatase (Dako) 1:1000 and nitro blue tetrazolium chloride and 5-bromo-4-chloro-3-indolyl phosphate (NBT/BCIP) (Roche) was used to detect bound antibody.

## Results

### 3.1 Effect of Kozak sequence

The translation initiation sites of HSA in the vectors G1 and H1 have semi Kozak sequences, CACCATG and CCATG, respectively, and are efficiently expressed in COS-7 (Figure 2) cells and horse lung cells but to a low extent in horse skin cells and poorly in duodenum and kidney cells (Figure 3). The wild type translation initiation site of HSA was replaced by the Kozak consensus sequence, GCCACCATG, in the two vectors W1 and V1 containing the intron A to obtain the vectors W2 and V2. In COS-7 cells V2 shows slightly more expression than V1 but the expression of W2 was diminished as compared to the W1 parent (Figure 2). W1 and V1 were expressed to a low level in cells from lung and to a very low level in skin, kidney and duodenum cells (Figure 3). This was significantly changed in W2 and V2 as the insertion of the Kozak sequence increased expression in all four horse cell lines as compared to the parent vectors W1 and V1. In the skin and kidney cells the expression from W2 and V2 reached similar levels to that of G1 and H1 that have a semi Kozak sequence. In the duodenum cells the expression from both W2 and V2 exceeded the G1 and H1 expression. In the lung cells the V2 showed similar level of expression as the G1 and H1 but W2 slightly higher expression (Figure 3).

### 3.2 Effect of intron A

The vectors G and H1 that do not contain intron A are similarly expressed in all the cells. Insertion of intron A into H1 to make H2 resulted in poorer expression of H2 both in COS-7 cells (Figure 2) and in all four horse cells as compared to the parental vector H1 (Figure 3). Despite of containing Intron A the W1 and V1 vectors show less expression than G and H1 in all the cells. This is presumably because of the lack of a Kozak sequence, as W2 and V2 vectors that have both intron A and a Kozak sequence show similar or higher expression than G and H1 in the horse cells (Figure 3).

The pBudCE4.1/lacZ/CAT plasmid was used as a control for the transfection. In the skin and lung cells the CAT expression was similar showing that similar amount of DNA was transfected and similar amount of cells were harvested from each well. However, the CAT expression was hardly or not detected in the kidney and duodenum cells (Figure 3).

## Discussion

Seven different mammalian expression vectors were compared for their ability to drive high levels of HSA protein expression in four different primary horse cells and COS-7. Two of the vectors, G1 and H1 with the HSA gene have been tested for DNA vaccination in horses, and both induced low immune response [23]. In order to develop vectors that have a significant expression in horse cells we investigated the effects of Kozak consensus and intron A sequences on the levels of expression of the HSA gene.

Sequences flanking the AUG initiation codon within mRNA have been shown to be important in recognition of the initial AUG. The consensus sequence surrounding the start codon is known as the Kozak consensus sequence, GCCA/GCCAUGG. The G at position +4 and A/G at position -3 of the start codon are especially important because lack of these bases causes reduction in efficiency [22,24]. This translation initiation signal directs the ribosomes to initiate protein synthesis from mRNAs. It is postulated by the scanning mechanism of initiation that the 40S ribosomal subunits enters at the 5' end of the mRNA and scans downstream until it comes across the first AUG codon. Initiation by ribosomes will start at the first AUG codon, but if there is a weak or no Kozak consensus sequence some ribosomes bypass and continue to scan downstream until another AUG start codon has been encountered. This is called leaky scanning [25]. In horses the Kozak sequence is commonly found as an initiation signal for gene translation as in other vertebrates [21]. For equine arteritis virus suboptimal intraleader AUG and not an optimal Kozak sequence has been shown to be critical for virus replication [26].

Although the HSA in the vectors G and H1 have only semi Kozak sequences (bold), TT**CACCATG**A and AATT**CCATG**A respectively, they are efficiently expressed in COS-7 (Figure 2) cells and horse lung cells but to a low extent in skin cells and poorly in duodenum and kidney cells (Figure 3). The vectors W1 and V1 do not have a Kozak consensus sequence and were expressed to a low level in cells from lung and to a very low level in skin, kidney and duodenum cells (Figure 3).

The Kozak consensus sequence, GCCACCATG, was inserted into the W1 and V1 vectors that already contained intron A. This significantly changed the expression of the progeny vectors W2 and V2 in all horse cell lines (Figure 3). No convincing effect was seen in the COS-7 cells (Figure 2).

Leaky scanning is a likely reason for the bands of lower molecular weight than 73 kDa HSA band seen in the blots (Figure 2 and 3) as their sizes match with the positions of AUG codons downstream in the HSA gene. However, insertion of the Kozak sequence is not reflected in less pronounced extra bands, at least not in vectors W1 and W2.

One of the critical elements to have on an expression vector is an intron to increase the efficiency of transcription [19]. The removal of the introns by the spliceosome influences the mRNA processing like initial transcription of gene, editing and polyadenylation of the pre-mRNA and nuclear export, translation and decay of the mRNA product [20]. The intron A was amplified from vector V (VR1012) and inserted into H1 to make H2 resulting in diminished expression of the HSA gene in the horse cells and COS-7. This could be because the intron A is not in the same position as in the original vector since there are 111 additional nucleotides between the CMV promoter and intron A (Figure 1). Attempts to amplify and insert the intron A together with the CMV promoter were unsuccessful.

Despite of intron A the W1 and V1 vectors were expressed less in comparison to the pcDNA vectors G1 and H1. This can probably be accounted for by the lack of a Kozak sequence as G1 and H1 contain a semi Kozak and introduction of a whole Kozak into W1 and V1 made a significant difference in their expression. To our knowledge no functional studies have been conducted so far that demonstrate the significance of full consensus Kozak sequences for protein expression in horse cells.

We are especially interested in Th1 directing the immune response of horses in the attempt to develop a vaccine against insect bite hypersensitivity (IBH). IBH is recurrent seasonal dermatitis of horses, an allergic reaction to bites of *Culicoides *spp., (biting midges) [27-30]. IBH is especially common in Icelandic horses exported to the continent as *Culicoides *spp. are not indigenous to Iceland [31].

We only obtained low level immune response in horses and not sufficiently Th1 focused by repeated intramuscular and intradermal injection with the HSA gene on the G1 and H1 vectors [23]. According to this and the results of others in the field some combination of immunizations, different types of vectors and/or protein boost, might be the approach to consider [5,6,32]. The HSA on W2 with a whole Kozak and intron A is expressed to a considerably greater extent than the G1 and H1 vectors especially in horse duodenum cells. Therefore we conclude that W2 could be one of the candidates for the development of Th1 focusing vaccines of horses.

## Competing Interests

 The authors hereby declare that they have no competing interests.

## Authors contribution

GO:  participated in the design of the study, carried out the molecular cloning, sequencing, cell tranfections, WB analyses and drafting the manuscript.  VS:  participated in the design of the study, molecular cloning, cell transfections and drafting the manuscript.  SI:  participated in the design of the study and drafting the manuscript.  EM: participated in the design of the study and drafting the manuscript.  ST: participated in the design of the study, cell cultivations, WB analyses and drafting the manuscript.    

All authors read and approved the final manuscript.  
